# A Review of National-Level Adaptation Planning with Regards to the Risks Posed by Climate Change on Infectious Diseases in 14 OECD Nations

**DOI:** 10.3390/ijerph10127083

**Published:** 2013-12-12

**Authors:** Mirna Panic, James D. Ford

**Affiliations:** 1 Institut national de santé publique du Québec, 190 boulevard Crémazie Est, Montréal, Québec, H2P1E2, Canada; 2 Department of Geography, McGill University, 805 Sherbrooke Ouest, Montréal, H3A2K6, Canada; E-Mail: james.ford@mcgill.ca

**Keywords:** climate change, adaptation, public health, infectious disease, high income nations, adaptation evaluation

## Abstract

Climate change is likely to have significant implications for human health, particularly through alterations of the incidence, prevalence, and distribution of infectious diseases. In the context of these risks, governments in high income nations have begun developing strategies to reduce potential climate change impacts and increase health system resilience (*i.e.*, adaptation). In this paper, we review and evaluate national-level adaptation planning in relation to infectious disease risks in 14 OECD countries with respect to “best practices” for adaptation identified in peer-reviewed literature. We find a number of limitations to current planning, including negligible consideration of the needs of vulnerable population groups, limited emphasis on local risks, and inadequate attention to implementation logistics, such as available funding and timelines for evaluation. The nature of planning documents varies widely between nations, four of which currently lack adaptation plans. In those countries where planning documents were available, adaptations were mainstreamed into existing public health programs, and prioritized a sectoral, rather than multidisciplinary, approach. The findings are consistent with other scholarship examining adaptation planning indicating an *ad hoc* and fragmented process, and support the need for enhanced attention to adaptation to infectious disease risks in public health policy at a national level.

## 1. Introduction

Climate change has been identified as the biggest global health threat this century, with a variety of direct and indirect impacts projected [1]. Changing temperature and precipitation regimes are expected to increase the probability, duration and severity of extreme weather events (e.g. flooding, storms), to increase the risk and incidence of some infectious diseases (e.g., malaria), and to affect food and water security [2,3,4,5,6,7,8,9,10,11,12]. These impacts will be exacerbated by social, economic, demographic, and other environmental stressors, including poverty, water and air pollution, land use change, economic development, population growth, and changing migration patterns [9,13,14,15,16], with the elderly, children, and other socially and economically disadvantaged populations particularly vulnerable [1]. While developing nations, particularly the Least Developed Countries (LDCs) and Small Island Developing States (SIDS), are widely believed to have the highest vulnerability to climate change, high income nations are also vulnerable, a fact evident in morbidity and mortality documented during recent climate-related disasters (e.g., the 2003 European heat wave, hurricanes Katrina and Sandy, record-breaking Australian wildfires, Alberta flooding) [17,18,19,20,21,22,23,24]. 

In light of the risks posed by climate change, and existing vulnerabilities to climate-related health outcomes, adaptation has emerged as a key focus of climate policy. Adaptation refers to policies, measures and strategies designed to reduce climate change impacts and foster resilience, a concept analogous to the population health notions of primary, secondary and, at times, tertiary prevention [13,19,23,25]. Within a climate change adaptation context, primary prevention would imply reducing potential community exposures linked with climate change, such as eliminating potential mosquito breeding sites to reduce the vector’s range expansion potential, while secondary prevention would consist in the detection and treatment of existing disease before substantial morbidity or mortality is incurred (e.g., the application of biocides to limit disease transmission in high risk areas, as defined by integrated surveillance data). Potential adaptations are hence diverse and range from specific interventions to address a known disease risk to building adaptive capacity, which is defined as the potential of a system to respond to change. Such actions may be undertaken at various scales, and may be reactive or anticipatory in relation to climate change impacts [26]. 

The majority of research and policy debate on health adaptation has focused on identifying risks posed by climate change, estimating their magnitude and extent, describing potential patterns of exposure and vulnerability, and highlighting priorities for intervention [12,27,28]. In some cases, nations have developed strategic priorities for adaptation [29,30]. While some studies have evaluated these adaptation plans as part of regional-scale analyses of the current status of adaptation in general [29], few studies have examined national-level adaptation planning specifically with respect to their adequacy in managing climate-related health risks. Thus for infectious diseases—an area of particular concern for public health professionals in a changing climate [12,31]—while there is a large and growing body of scholarship assessing vulnerability to future impacts, our understanding of how adaptation is being prioritized is limited [32,33]. However, such assessments of adaptation are important if we are to identify gaps in problem framing in order to pave the way towards a more robust practice of adaptation strategy development [29].

In light of an absence of research examining how adaptation figures in health policy, this paper evaluates national-level planning for adaptation with regards to infectious disease-related impacts of climate change in OECD nations. Specifically, we identify the types of adaptations proposed in adaptation plans of national agencies and governments, and examine whether there are gaps in current public health planning by comparing with recommendations for infectious disease adaptation in the peer reviewed literature. Our focus on national-level planning reflects the role of national governments as a central pivot for adaptation, whether it be by catalyzing interest in adaptation, determining policy priorities, or allocating resources and support [32,34,35]. 

## 2. Methods

### 2.1. Scientific Literature Selection

To identify best practices proposed within the scientific literature to respond to climate change-related infectious disease risks, an assessment of peer-reviewed literature was conducted. Five databases were searched (PubMed, MEDLINE, EMBASE, Web of Knowledge, Scopus) using database-specific search strategies (see Supplementary Materials for the full search process). Reference lists were scanned for additional citations. Searches were last updated on 17 April 2013, with no time limit set on the search. All available peer-reviewed literature in English, French and Spanish proposing public health adaptations to infectious disease risk of climate change was reviewed, with 54 articles retained for full review (see Supplementary Materials). Articles addressing climate change impacts on infectious disease dynamics were excluded if they exclusively addressed risks, or recommended adaptations, in relation to developing-country or middle-income country settings. These articles were excluded due to potential limited relevance to OECD-settings. 

### 2.2. Adaptation Plan Selection

To assess adaptation planning by OECD nations for infectious disease risks, national adaptation plans, as well as official Public Health and Health Ministry reports addressing adaptation or public health response to health threats of climate change, were selected and analyzed for a sample of fourteen OECD-member countries: Australia, Belgium, Canada, Chile, France, Ireland, Luxembourg, Mexico, New Zealand, Slovenia, Spain, Switzerland, the United Kingdom, and the United States. Selection sought to capture a diversity of nations for whom information was available in languages spoken by the research team (English, French and Spanish). The OECD-country sample was based on the availability of national-level planning documents published in the abovementioned languages. To be included in the review, plans and official documents had to: substantively focus on adaptation, either through the promotion of specific interventions to minimize threats from infectious diseases or description of approaches to build adaptive capacity in the health sector; have climate change as an overarching rationale; focus on the national scale; and specifically address risks from infectious diseases, which were defined as any zoonotic, vector-borne, food-borne or waterborne diseases which may be directly impacted or exacerbated by climate change (see Table 1). 

**Table 1:** Climate-Dependant infectious diseases and sample countries likely to experience health hazards linked to changes in disease exposure. Inspired from ECDC 2010 [36].

Disease type	Disease	Environmental factors impacting disease dynamics	Countries likely to be affected
Mosquito-borne diseases	Malaria	Increased average temperatures	Australia, New Zealand, Chile, Southern Europe
	West Nile Virus	Increased average temperatures, drought	USA, Southern Europe, Canada, Australia, New Zealand, Chile
	Dengue, Chikungunya fever, Yellow fever	Increased average temperatures	New Zealand, Mediterranean region (coastal areas in Spain, Portugal and France), Chile
Tick-borne diseases	Lyme borreliosis, tick-borne encephalitis,	Increased daily precipitation, humidity, changed patterns of seasonal precipitation, Increased average temperatures, extreme heat	Northern Europe, Canada, USA
Waterborne diseases	Sewage and sanitation: Vibrio vulnificus and Vibrio cholera, E.Coli, Campylobacter, Salmonella, Cryptosporidium, Giardia, Yersinia, Legionella	Increased rainfall and storm frequency, flooding, landslides, increased average temperatures, extreme heat episodes	All countries
Food borne diseases	Salmonellosis, campylobacteriosis	Extreme rainfall, flooding, increased average temperatures, increased frequency of extreme heat, changed seasonal patterns	All countries

National-level plans and official documents were identified through a web-based search of official public health agency and governmental websites using search terms and inclusion criteria presented in Table 2. The search was supplemented through country-specific Google searches, document reference and citation tracking, and key word searches in specialized policy databases, including: Bandolier, Latin-American and Caribbean Center on Health Sciences Information, National Health Services (NHS), University of York Centre for Reviews and Dissemination, Organization for Economic Co-operation and Development, and the WHO Global Health Observatory (GHO). We used the method of Furgal *et al.* [37], advanced in a climate change context by Poutiainen *et al.* [38], to manage the large number of hits obtained through Google, with each of the first 30 hits for a single search reviewed for inclusion in the study. After 30 hits, each second result was reviewed until twenty consecutive irrelevant results were found, at which point the search was stopped. Typically, this led to the review of the first one hundred hits. 

**Table 2:** Key words, inclusion and exclusion criteria for grey literature document retrieval and selection.

**1. Key Word Search**	**Terms used:**
1.1 English	“climate change”, “global warming” AND/OR “infectious disease”, “communicable disease”, “zoonos*s”, “waterborne disease”, “food*borne disease”, “vector*borne disease”
1.2 French	“changement climatique”, “réchauffement climatique” AND/OR “maladies infectieuses”, “maladies à transmission vectorielle”, “maladies émergentes”, “maladies diarrhéiques”, “maladies d’origine hydrique”, “intoxications alimentaires”, “maladies d’origine alimentaire”, “zoonoses”
1.3 Spanish	“cambio climatico”, “calienmente global” AND/OR “enfermedades transmisibles”, “enfermedades infecciosas”, “zoonosis”, “enfermedades de transmisiόn vectorial”, “enfermedades transmitidas por el agua/ por los alimentos”, “enfermedades emergentes”
**2. Inclusion Criteria**	**Exclusion Criteria**
English, French, Spanish documents	Non-English, French, Spanish
Technical documents, Adaptation Plans, National Reports, Adaptation Assessments, Vulnerability Assessments containing recommendations.	Editorials, Meetings and Conferences, Abstracts
Human Adaptation to Climate change	Natural and/or biodiversity focus, focus on climate change mitigation
Practical focus (detailing adaptation activities or actions)	Enumeration and assessments of vulnerabilities only, description of the problem and potential hazards only, conceptual documents only.
OECD Nations	Non-OECD Nation

### 2.3. Analysis

The assessment of peer-reviewed publications was qualitative in nature: we aimed to identify and list specific adaptations recommended. This list was subsequently compared to adaptation policies proposed within national adaptation planning documents to identify gaps between needs identified within the literature and the policies proposed at the national-level. By listing, comparing, and contrasting adaptations this way, we aimed to assess whether and to what extent recommendations formulated in the literature were integrated into national adaptation plans. 

## 3. Results and Discussion

### 3.1. Infectious Disease Adaptations Proposed in the Literature

Adaptations to infectious disease risks proposed in the peer-reviewed literature are summarized in Table 3, and can be classified in six overarching categories: reduction of occupational health risks, adaptations to risks from vector-borne, waterborne and food-borne diseases, improved monitoring and surveillance, and capacity-building. 

**Table 3:** Recommended public health adaptation strategies in the peer-reviewed literature.

Topic	Recommendation
**Occupational Health**	Identify vulnerable professional groups [39]
	Develop suitable protective clothing and gear [39,40]
	Heighten workplace awareness of infectious disease risks [39]
**Waterborne Disease**	Ensure adequate water supply and quality [18,40,41,42,43,44,45,46,47,48,49]
	Increase drinking & recreational water quality monitoring in relation to specific climate and weather patterns (e.g.,: increased precipitation) [42,45,50,51]
	Create advisory platforms and improve outreach [42,43]
	Improve watershed protection and management [18,40,42,46,52]
	Develop new drug therapies for waterborne diseases [42]
	Develop membrane filters to address cyanotoxicity [43,53]
	Consider water-pathogen source placement (e.g.,: cattle farms) [18,49]
	Improve wastewater disposal and municipal water systems [51]
	Involve nursing staff in community microbiological water testing [51]
**Food-borne Disease**	Enforce appropriate food production, monitoring and handling standards [48,50,54,55,56,57]
	Provide public education campaigns to promote good practices in food preparation [42,54,55]
	Increase monitoring of preparation practices within institutions [54]
	Develop a national integrated system of food tracking from farm to fork [45,54]
	Incentivize the local production of food [54]
	Intensify existing food safety programs during warmer periods and optimize food disinfection protocols [43,58]
	Provide freezer programs for hunting communities [47]
**Vector-borne disease**	Develop vaccines for human and animal host-species [1,18,40,42,59,60,61,62]
	Link human health and veterinary sciences in public health practice [59]
	Create or strengthen animal and wildlife sentinel surveillance systems [45,57,59]
	Implement preventive strategies for sustainable livestock production [59]
	Harmonize case reporting across regions and national boundaries [63]
	Improve vector control [1,18,40,42,55,57,60,61,64,65,66]
	Strengthen preparedness and response to extreme weather events [64]
	Encourage individual level adaptations such as the use of mosquito nets [1,40,47,55,57,60,62,64,67,68]
	Domestic water tank screening, urban runoff capture and improved urban drainage systems [18,54,60]
	Incorporate fly screens in construction norms [54]
	Implement adequate goods-importation laws and monitoring [42]
	Supplement current surveillance programs with additional surveillance sites for monitoring [56,62]
**Surveillance **	Further develop genomic surveillance [69]
	Develop novel disease and vulnerability indicators [39,56,61,67,70]
	Expand disease tracking surveillance programs [1,18,42,50,52,56,57,61,66,71,72,73]
**Surveillance**	Collect data on environmental risks to perfect integrated monitoring and forecasting systems [1,18,41,51,54,56,61,62,63,64,67,69,72,74,75,76,77,78,79]
	Collect data on vulnerabilities and identify vulnerable populations [18,41,42,54,61,72,75,80]
	Perfect early-warning and syndromic surveillance systems integrating environmental, ecological, veterinary and epidemiological data [18,41,42,55,57,61,62,64,70,81,82]
	Ensure adequate data collection and data quality [1,47,57,64,65,70,72,74,81]
	Develop the use proxy measures and interpolation when data may be unavailable [74]
	Develop spatial analysis technologies with greater integrative analysis capabilities than current GIS software [52,57,67,73,75,79]
	Increase the ability to share data and information across jurisdictions [42,57]
	Improve the timeliness of access to laboratory testing and its results [42]
	Integrate multidisciplinary knowledge in surveillance and risk assessments [47,56,57,61,67,72]
	Integrate community participation in surveillance [19,61,70]
**General Strategies, and Capacity Building**	Provide education about ID risks of CC, individual adaptation measures and/or mainstreaming in existing health promotion programs [1,41,47,51,57,61,64,66,83]
	Provide regular (and updated) workforce training [41,42,57,73,84]
	Prepare health care workers and public health professionals to potential ID risks of CC [39,42,61,80,81,82,84]
	Incorporate ID risks of climate change in medical and university training curricula and create new training programs [1,41,42,50,59,61,81,85,86]
	Develop and validate new diagnostic tests protocols [18,59,69]
	Involve stakeholders and the media to increase awareness and identify alternative adaptation options [19,54,59,73]
	Build capacity by increasing infrastructure and research capabilities, the provision of adequate funding, equipment and trained staff [57,59,64,73,80,81]
	Focus adaptation efforts to vulnerable communities [18,52,57,64,80]
	Improve vaccination coverage and public immunization campaigns [57,60,61,64,67]
	Cooperate with relevant sectors : meteorology, environment, urban planning, hydrology, agriculture [56,61,64,78,79,80]
	Emphasize adaptive management, constant monitoring and evaluation, and the implementation no-risk options [54,61]
	Improve access to preventive and primary care [1,42,50,57,61,80]
	Improve laboratory infrastructure and testing capabilities [42]
	Conduct cost-effectiveness analyses of proposed adaptation strategies [76]
	Improve forecast modeling [55,62,78,82,87,88,89]
	Assess stakeholder conceptualizations and approaches to health [72,90]
	Evaluate opportunities for policy intervention (effectiveness, desirability, feasibility, urgency, equity, durability) with the use of scenarios [72]
	Create community and stakeholder partnerships, encourage social involvement and foster social networks. [19,61,72,90]

It is noteworthy that some recommendations were applicable to health risks of climate change in general, and were presented as such within the reviewed publications: they were included here because of their specific relevance to infectious-disease risks. 

At a general-level, emphasis is placed on the unequal distribution of impacts and consequent need to identify and target vulnerable populations through coordinated outreach campaigns within all six adaptation categories e.g., [18,39,42,43,47,52,54,55,56,57,61,64,67,70,80]. Such campaigns may be aimed at incentivizing individual-level responses, such as the wearing of protective clothing outdoors, the adequate cooking and washing of food products, and the use of bed nets; or to educate individuals and communities more generally with regards to disease etiology and potential symptoms. The necessity of individual-level adaptations and, more broadly, the use of low-cost and low-technological solutions such as mosquito nets and water filters, as effective public health measures to respond to hazards associated with climate change has been recognized as “tried and tested” best practice from the very beginnings of public health adaptation research [47]. 

While individual adaptations remain crucial, the need for the community-based adaptations to health risks of climate change, as well as for the fostering of sustainable stakeholder partnerships, has been increasingly highlighted in the literature [19]. The creation of multidisciplinary teams of health professionals and the integration of knowledge from a variety of research areas and applied sciences in public health practice has also emerged as an essential component of recommendations for fostering long-term sustainable adaptation. These trends reflect a growing recognition in the infectious-disease literature of the complex nature of risks related to climate change—which may themselves be considered outcomes of ecological, epidemiologic, and socio-economic interactions—and of a need for multidisciplinary and cross-sectoral cooperation [18,41,42,55,61,62,64,70,81,82].

The need for trans-disciplinary collaboration is increasingly apparent within sectors aiming to develop novel approaches for the elaboration of integrated surveillance systems. Emerging threats from vector-borne diseases have required public health professionals to increasingly move away from of conventional conceptualizations of pathogen-human-host transmission scenarios and incorporate advanced notions of ecology, entomology and veterinary sciences to evaluate risks of disease spread. The development of methods to assess potential impacts of mosquito-borne disease [64,91] is a recurring example of potential multi-sectoral challenges brought forth by climate change. The need for integrated surveillance systems is highlighted in the scientific literature by clear research needs and information gaps which would be impossible to tackle without substantial data and knowledge sharing across disciplines and geographical boundaries. 

### 3.2. National-Level Adaptation Planning for Infectious Disease Risks of Climate Change

The majority of the policy documents reviewed here focus broadly on adaptation to climate change across different public sectors (Table 4), with some technical reports targeted at a single sector or organization undertaking the implementation or coordination of adaptation strategies. Primary examples of the latter include adaptation planning documents from the United Kingdom and the United States. The official documents reviewed were supplemented by information available online, to account for potential plan updates and revisions. Ireland, Slovenia and Luxembourg have not yet published their respective adaptation plans, but have indicated that they will be publically available in 2013/14. A preparatory workshop on health system adaptation needs is available on the Climate Change office of the Republic of Slovenia website. Likewise, a draft of the adaptation plan for Luxembourg could be found, though the official version is not yet available for public consultation. These preliminary documents were not included in this review as they are still undergoing modifications and may not be representative of the nation’s official policy position.

**Table 4:** National-Level adaptation planning documents reviewed in this study.

Country	Adaptation Plan	Drafting Body
**Australia**	National Climate Change Adaptation Framework [92] National Climate Change Adaptation Research Plan: Human Health (Update: 2012) [93,94]	Council of Australian Governments National Climate Change Adaptation Research Facility
**Belgium**	Plan National Climat de la Belgique 2009–2012 [95]	Commission Nationale Climat: Groupe de Travail Politiques et Mesures
**Canada**	Climate Change Impacts and Adaptation: A Canadian Perspective [96] From Impacts to Adaptation: Canada in a Changing Climate 2007 [97]	Climate Change Impacts and Adaptation Directorate Government of Canada
**Chile**	National Climate Change Action Plan 2008–2012 [98]	National Environmental Commission Gobierno de Chile
**France**	L’adaptation de la France au changement climatique [99] Plan national d’adaptation de la France aux effets du changement Climatique 2011–2015 [100]	Observatoire national sur les effets du réchauffement climatique Ministère de l'écologie, du développement durable, et de l’énergie
**Spain**	Cambio Global España 2020/50. Cambio climático y salud [101]	Centro Complutense de Estudios e Información Medioambiental & Instituto Sindical de Trabajo, Ambiente y Salud & Sociedad Española de Sanidad Ambiental
**Switzerland**	Adaptation aux changements climatiques en Suisse: Objectifs, défis et champs d’action Premier volet de la stratégie du Conseil fédéral du 2 mars 2012 [102] Les changements climatiques et la Suisse en 2050: impacts attendus sur l'environnement, la société et l'économie [103]	l’Office fédéral de l’environnement ProClim—Forum for Climate and Global Change
**UK**	Department of Health: Climate Change Plan [104] Health Effects of Climate Change in the UK 2012 Current evidence, recommendations and research gaps [105] The National Adaptation Programme [106]	Central Office of Information for the Department of Health Health Protection Agency DEFRA
**USA**	Climate Change and Health program website Progress Report of the Interagency Climate Change Adaptation Task Force: Recommended Actions in Support of a National Climate Change Adaptation Strategy 5, October, 2010 [107] HHS Climate Change Adaptation Plan [108]	Centers for Disease Control The White House Council on Environmental Quality Department of Health & Human Services

The majority of documents reviewed contained at least one suggestion for potential adaptation that may be undertaken in relation to changing infectious-disease dynamics with climate change (Table 5). However, the specificity of the proposed adaptations varied substantially between plans. Some only broadly outlined public health principles for adaptation: for example, current recommendations in Switzerland’s adaptation plan [102] largely focus upon the importance of multidisciplinarity in tackling climate change-related health risks, sharing of data and information across sectors and the integration of “new risks” in current public health strategies. The adaptation plan does not, however, describe any detailed objectives that must be attained to support the realization of these principles. Specifically, it does not mention how professionals within different scientific disciplines, or governmental bodies, ought to be cooperating to optimize knowledge sharing. Interestingly, the document doesn’t explicitly name infectious agents or diseases which may become an emerging or amplified threat in relation to climate change (though it mentions threats brought forth by the propagation of certain vectors), and does not detail current gaps in public health strategies to address such risks. A broad assessment of potential future infectious disease risks in relation to climate change is available in another national-level risk assessment [103]. In contrast, other plans, such as that proposed by the Australian government [93], go considerably further in planning development and identify the ministries, organizations and stakeholders which are to carry out and evaluate the proposed strategies; all of these aspects are of key importance in creating readiness for adaptation [35,109].

Wide variations in the nature of adaptation planning for infectious diseases are evident across the 14 countries analyzed, even in cases where countries are situated within neighboring regions facing similar impacts. For example, despite the fact that the South Pacific region is likely to be strongly impacted by changing vector ranges and environmental conditions [110,111], New Zealand’s national adaptation plan only outlines broad suggestions of methods to alleviate future health risks of climate change and focuses primarily on the benefits of mitigation. For example, New Zealand’s adaptation plan presents “walking, cycling and taking public transport” as individual-level “adaptations” meant to increase physical activity and diminish one’s carbon footprint. In general, scientific publications classify such measures as health co-benefits of mitigation strategies [48]. Though the plan includes a section examining New Zealand’s vulnerabilities to infectious diseases (e.g., increasing incidence of Dengue and Ross River virus), none of the proposed adaptation measures directly address the risks related to evolving infectious disease dynamics. In contrast, Australia has created the National Climate Change Adaptation Research Facility to support adaptation science and complement a regularly updated Human Health Adaptation Plan, in which priorities for action are revised in light of emerging evidence and highlighted needs. 

European countries are also far from homogenous in their approaches to tackling infectious disease risks. However, they benefit from the overarching support of the European Center for Disease Control, which has played an important role in information sharing and the creation of multidisciplinary support structures and professional networks (ex: VBORNET) [56]. This is reflected in the planning of several EU states. Spain’s adaptation plan, for instance, comprises an in-depth discussion of potential infectious disease hazards and lists several suggestions to reduce future threats, such as conducting spatial assessments of risk, the development of early-warning systems and vector monitoring, increased surveillance (particularly in areas of high circulation, such as airports), the training of national climate change and entomology expertise, vector control, and the provision of educational outreach campaigns for health professionals and the general public. This adaptation strategy is rooted within a national public health context that place significant emphasis on developing mosquito-borne disease surveillance, and is hence familiar with the complexities inherent to such a process. Similarly, the French adaptation plan also discusses specific health risks of climate change, and comprises broader strategy recommendations, such as the establishment of a new climate change adaptation research group. Additionally, concrete actions are proposed to aid in the integration of spatial analysis in current methods of vector and pathogen reservoir surveillance, as well as to improve food refrigeration and water treatment, among several others. Each adaptation is accompanied by a target year of implementation and a list of ministries and partners involved in its deployment and coordination, a process substantially more advanced than that of countries such as New Zealand. Lastly, the Australian government has created a Department of Climate Change to facilitate the coordination of activities at the national level [109]. This highlights a potentially worrisome contrast in climate change planning: countries with more experience in implementing adaptation strategies may be more aware of existing vulnerabilities, and may hence be more likely to prioritize remediating to known deficiencies in current national policy development. 

**Table 5:** Development of strategies and methods for adaptation—adaptation plans by country.

Country	Awareness of CC Impact on ID Dynamics	Evidence for Adaptation Strategies/Plans in PH	Infectious Disease-Specific Adaptation Measures
Australia	√	√	√
Belgium	√	√	
Canada	√	√	√
Chile	√	√	√
France	√	√	√
Ireland	√		
Luxembourg			
Mexico	√		
New Zealand	√		
Slovenia	√	√	
Spain	√	√	√
Switzerland	√	√	√
UK	√	√	√
USA	√	√	√

Canada stands-out in terms of breadth and completeness of qualitative national reports, in particular in relation to the 2008 national assessment of vulnerability and adaptive capacity in the health sector, led by Health Canada [112]. This report details area-specific health hazards of climate change and identifies potential needs for adaptation. As it is the case for Australia, the heightened quality of the work made available by Health Canada and the Public Health Agency of Canada may be directly related to the department’s emphasis on the assessment and production of rigorous scientific publications investigating the links between policy, climate and health [42,84]. Additionally, information regarding potential adaptation research funding (grants) offered by the Public Health Agency of Canada and Health Canada for climate change adaptation is advertised and easy to find on the agency’s official website. Over the period from 2011 to 2016, Canada has allocated $149 million to support adaptation implementation and capacity building [113]. Nonetheless, Canada has yet to publish a national-level adaptation plan. Similarly, American initiatives, though supported by an Interagency Climate Change Adaptation Task Force of the White House Council on Environmental Quality and a CDC climate adaptation initiative, remain primarily on a state by state basis. An overview of adaptation planning activities occurring in American states can be found in Smith *et al.* and Bierbaum *et al.* [114,115].

### 3.3. Classification of Adaptation Options and Between-Plan Comparisons

National adaptation plans are meant to be frameworks or guidance documents for individual agencies to structure the prioritization of their adaptation needs. The inherent goal of public health adaptation, and adaptation planning by proxy, is the reduction of specific vulnerabilities to the potential effects of climate change. In order to ensure that vulnerabilities are indeed being addressed, the need for mechanisms to evaluate the degree of rigor associated with adaptation planning and tracking adaptation planning outcomes has become apparent [29]. 

To assess the comprehensiveness of adaptation planning, the selected documents (Table 4) were assessed and classified using a typology presented by Preston *et al.* (2011) [29], focusing primarily on the planning processes by which adaptation actions may be selected for subsequent implementation. This typology aims to address the lack of systematic indicators illustrating the range of potential activities that might be expected from robust adaptation planning documents, or the aspects that ought to be considered when creating guiding frameworks for future adaptation actions. It broadly groups the planning processes in two overarching objectives of adaptation planning: the building of adaptive capacity and the delivering of adaptation actions [29].

Within the scope of this review, the typology proposed by Preston *et al.* (2011) [29] was used to facilitate comparison between plans with respect to the broad adaptation planning processes adopted within them to detect preferences and emphases in conceptualizations of health adaptations among national institutions. This classification allowed for an analysis of overarching themes and approaches to problem framing within individual adaptation plans to identify potentially unaddressed (or under-developed) aspects of adaptation planning (Table 6). These unaddressed aspects were further informed by best-practice measures proposed in peer-reviewed scientific literature (see Section 3.1.). 

It becomes immediately apparent, through the use of this classification typology that proposed adaptations are primarily concerned with sectoral (90%), rather than integrated, multidisciplinary adaptations implicating different agencies, jurisdictional levels or stakeholders. For example, Switzerland’s proposed plan explicitly divides the objectives of the adaptation plans in broad sectors (“*Adaptations Sectorielles*”): water management, natural disaster management, agriculture, forestry, energy, tourism, biodiversity, health, territorial development. However, this plan makes explicitly clear that single impacts of climate change (ex: heat waves) will impact multiple sectors at once (Figure 2.1., page 8 of the plan, and throughout Section 2 which details the country’s potential vulnerabilities to climate change), and that different governmental bodies will need to work “hand in hand” (page 9) to tackle them. It is not specified how this will be achieved, nor are these linkages revisited in section 3, which details the proposed sectoral adaptation strategies. 

**Table 6:** Specific types of adaptations to infectious disease risks of climate change proposed within reviewed documents and classification of adaptation options according to Preston *et al.*’s (2011) [29] proposed typology.

Adaptations Proposed	Classification of Adaptation strategies	Consideration of Vulnerable Populations	Sectoral or Holistic	Initiative or Mainstreaming	Nation Proposing
Ensure that existing surveillance systems are sensitive and efficient enough to detect new threats in a timely manner	Avoiding or reducing the risks	No	Sectoral	Mainstreaming	UK, France, Spain, USA, Canada, Australia, Switzerland
Strengthen surveillance systems which currently lack the capacity to integrate zoonotic and environmental data in disease detection	Avoiding or reducing the risks	No	Holistic	Mainstreaming and Initiative if development of new knowledge	UK, France, Spain, USA, Canada, Australia, Chile
Ensure that surveillance systems have the capacity to detect vector-borne diseases whose range is suspected to change (integrate epidemiological and environmental data)	Avoiding or reducing the risks	No	Sectoral	Mainstreaming	UK, USA, Chile, France, Spain, Canada, Australia
Increase awareness of potential effects of climate change within the public health surveillance, public health planning, infectious disease and medical communities	Creating supportive social structures	No	Holistic	Mainstreaming	Chile, France, Canada, Australia, Switzerland
Integrate climate and precipitation data in forecast and predictive models for the purposes of public health intervention & Use the growing scientific evidence base to inform the preparedness and responsiveness	Gathering and sharing of new information & exploiting new opportunities	No	Sectoral	Mainstreaming	Chile, France, Spain, USA, Canada, Australia, Switzerland
Increase the capacity for or continue to ensure appropriate water sanitation and water quality monitoring	Avoiding or reducing the risks	No	Sectoral	Mainstreaming	New Zealand, UK, USA, France, Canada, Australia, Switzerland Spain
Conduct water-consumption hygiene outreach campaigns	Avoiding or reducing the risks	No	Sectoral	Mainstreaming	France, UK, Chile, Belgium, Australia, Spain
Strengthen food quality regulation and monitoring	Avoiding or reducing the risks & creating a supportive institutional framework	No	Sectoral	Mainstreaming	France, UK, Canada, Australia, Spain
Invest in strategies for vector control	Bearing the risk	No	Sectoral	Mainstreaming	Chile, Canada, Australia, Spain
Increase networking between sectors and jurisdictional levels	Creating supportive social structures	No	Holistic	Mainstreaming	Chile, UK, USA, Canada, Australia, Switzerland
Increase education and public outreach campaigns	Gathering and sharing of information	No	Sectoral	Mainstreaming	New Zealand, Spain, UK, France, USA, Canada, Australia, Switzerland
Improve resilience to climate effects for the most vulnerable in society	Bearing the risks	Yes	Sectoral	Mainstreaming	UK, USA, New Zealand, Canada, Australia, Spain
Carry out an economic assessment of preventive measures, as well as infrastructure and personnel needs	Gathering and sharing of information	No	Sectoral	Mainstreaming	Chile, Canada, Australia, Switzerland
Strengthen the capabilities of health personnel to address prevention and care of adverse effects caused by climate change	Gathering and sharing of information & Creating supportive social structures & Institutional framework	No	Sectoral	Mainstreaming	USA, Canada, Australia, Spain, France
Create a multidisciplinary expert group for planning, evidence assessment and the formulation of recommendations	Exploiting new opportunities & Gathering and sharing of information & Creating supportive social structures	No	Holistic	Innovative (though not a new concept, it is a new body)	France, USA (BRACE), Australia
Explicit statement regarding the need to evaluate implemented strategies	Gathering and sharing of information	No	Sectoral	Mainstreaming	USA, Canada, Australia, Switzerland, France
Fostering international cooperation	Gathering and sharing of information & Creating supportive social structures	No	Sectoral	Mainstreaming	Canada, Chile, Spain, Switzerland, France

Indeed, cooperation between government bodies (national and local levels, between ministries) was not explicitly highlighted in the majority of reviewed plans. This was unanticipated prior to the assessment of documents, as the need for formal coordination mechanisms across ministries and organizations dealing with environment, water, agriculture, urban planning and health care is widely highlighted in scientific literature on infectious disease adaptation [116], and adaptation more generally [35]. Such coordination mechanisms require involvement of multiple stakeholders from government and industry at all phases of adaptation planning and assessments, in an iterative fashion which allows policymakers to determine which strategies are working, which could be improved and the appropriateness of select measures in addressing changes in incidence and spread of infectious diseases [116]. 

The importance of local government participation in public health adaptation planning, as a complement to national policy, as well as the need for local integrated vulnerability assessments, have been explicitly highlighted within the Australian *National Climate Change Adaptation Research Plan for Human Health,* the Canadian *From Impacts to Adaptation document*, the French, American and Spanish Plans. The UK Adaptation Programme most explicitly addresses the question of local governance, by situating the concept within the national legislative framework: “An important framework for managing health risks to the local population is defined by the Health and Social Care Act 2012, which places an important focus on local planning and decision-making, led by Directors of Public Health”. As the importance of local adaptation and capacity-building has long been highlighted in climate change literature [27,35,62], this type of explicit policy framing ought to become standard practice in subsequent national planning endeavors. 

A similar proportion of plans (90%) were primarily concerned with mainstreaming, defined as the integration of climate change into ongoing public health projects and priorities focusing on health promotion and protection [32,62]. Hence, the framework used within the majority of national documents for prioritizing adaptation policies and programs to control hazards of climate change situates adaptation planning within existing basic public health functions such as surveillance, outbreak investigation and response, education, research, and trend analysis. The obligation to “upgrade” the scope of existing core public health functions to account for projections of changing climates and infectious disease dynamics is highlighted in nearly all of the reviewed documents [61,116]. This clear need for better surveillance and monitoring methodology is hence a central aspect of both the national adaptation documents reviewed, and the peer-reviewed literature. Within Preston *et al.*’s typology for classification of adaptation processes, such strategies would fall primarily under the purview of *gathering and sharing information*, though they may also entail the *avoidance or reduction of risks* through upgrades to existing surveillance infrastructure. This is primarily true of arthropod-borne disease surveillance which may require the installation of mosquito trapping devices, sentinel surveillance systems, and more.

Additionally, the need to integrate environmental, ecological and veterinary variables into surveillance is widely recognized. Though most experts agree that spatial analysis will be important herein, increasing our ability to account for temperature, precipitation and water quality data in analysis and forecasting, few countries explicitly acknowledge the need to develop this resource. Across planning documents reviewed, a strong emphasis is placed upon the improvement of current surveillance systems. Very few plans however, specify how such improvements would be achieved (for example, by improving GIS software capabilities or through the integration of environmental and wildlife data to surveillance processes). It is also noteworthy that while waterborne and food-borne diseases are believed to pose the greatest risk in relation to climate change impacts in developed nations [28,58,117], limited emphasis is placed in adaptation plans upon adaptation measures in relation to such pathogens. At the very least this is a deficiency in the “*gathering and sharing of information*” planning process, a strategy otherwise widely prioritized within planning documents. This may reflect an inherent lack of awareness of potential effects of climate change on food and waterborne diseases amongst those drafting adaptation planning documents. 

As noted above, the majority of proposed adaptations were concerned with the *gathering and sharing of information*, as well as with *risk avoidance* (e.g., the development of forecast models and surveillance systems, vector control, improved water sanitation). Traditionally, these are public health strategies developed to maximize prevention and preparedness to uncertain disease risks: it is therefore logical that they are at the forefront of a planning process aimed at managing stochastic and complex disease dynamics. We must note, however, that though most plans broadly addressed the complex nature of environmental, ecological and epidemiological factors impacting infectious disease risks of climate change, the implementation of holistic approaches explicitly recognizing the need to integrate zoological, entomological, ecological, geographical and environmental sciences in health policy development is limited.

It is noteworthy that only five of the reviewed adaptation plans/documents (UK, Spain, Canada, Australia, USA) explicitly addressed the needs of vulnerable populations, while two explicitly highlighted the need to consider a vulnerable population approach in policy development without specifying how this would be achieved, despite the importance given to developing an understanding of the differential distribution of infectious disease impacts and of targeting interventions to the most vulnerable in the infectious disease literature [72]. Though we recognize that more resources need to be invested in the development of surveillance and monitoring tools as well as in health-related climate risk assessments to strengthen the capacity of the health system to respond to future stressors [20], this limited perspective on adaptation may prove inadequate without investments in initiatives to promote active community involvement [19] in resource and knowledge building, and without explicit considerations for vulnerable populations such as the elderly and local indigenous populations [72]. In response to recent studies in public health adaptation, the focus of the expert and policy dialogue has increasingly shifted towards community-based approaches to adaptation and vulnerability assessments in health [47].

## 4. Conclusions

In this paper, we have examined the extent to which national planning for adaptation is addressing the risks posed by climate change on infectious diseases, focusing on 14 OECD nations. Reviewing national-level adaptation plans and governmental public health reports, the study is one of the first to specifically focus on how infectious diseases are being addressed in adaptation, contributing to a rapidly growing scholarship identifying and characterizing the current state of adaptation more generally [29,32,33,118]. We acknowledge that the findings are preliminary, and represent a snapshot of adaptation activities at the national level. As such, our analysis does not capture adaptation planning taking place at lower levels of governance or that which is not reported on publically, and focuses on a limited number of nations because of practical nature of our sample. Though we limited our analysis and review of policy documents to those nation’s whose plans were published in English, French and Spanish, the documents selected for in-depth analysis correspond to a wide range of policy documents, which are representative of current adaptation planning across developed nations. Despite these caveats, the work raises a number of important points about the current status of adaptation planning for infectious diseases.

The comprehensiveness, extent and framing of adaptation planning within the OECD-nations examined are not homogenous and vary among jurisdictions. This is well illustrated, for example, by significant discrepancies on infectious disease adaptation planning between countries with similar risk profiles such as Australia and New Zealand. Despite the fact that New Zealand is likely to experience a change in the spread and incidence of infectious disease in coming years, the national adaptation plans only tangentially addresses the topic. In contrast, within a similar context, Australian adaptation planning documents present thorough, periodically revised guidelines to address the specific needs of their population. This reflects the government’s commitment to support local infectious disease adaptation, and interest in pursuing the climate change policy agenda, and illustrates the importance of government leadership in directing adaptation [119,120]. Differences in plan comprehensiveness and quality may furthermore be explained by findings in a recent study by Lesnikowski *et al.* [121], where the authors found that progress on adaptation is significantly related to policy commitments to engagement in international and national environmental governance. 

While this review primarily evaluates adaptation plans against peer-reviewed infectious disease literature with the goal of identifying gaps in current public health adaptation planning, in so doing it also raises deeper governance challenges. For example, where climate change adaptation is addressed in national level adaptation planning documents, the measures are typically “mainstreamed”, embedded within pre-established programs that were not originally constructed with climate change in mind. The most notable is the strong emphasis on the development and improvement of surveillance programs; a strategic priority rose both within select adaptation plans and peer-reviewed publications. Though mainstreaming through improved surveillance is appealing, the integration of environmental and social data in surveillance and forecasting requires more extensive departmental, organizational and disciplinary cooperation and coordination than the construction of traditional surveillance systems [122]. The mere information technology considerations necessary for cost-effective data sharing may be substantial. This difficulty is not explicitly addressed within the national adaptation plans reviewed. 

Undeniably, adaptation through mainstreaming is central in creating a pathway for the integration of climate change considerations into ongoing policy processes [123,124]. However, in the absence of innovative change and capacity building, mainstreaming may also diminish impetus for investing in targeted actions designed specifically to address climate change-related ID risks, whose effects will be progressive and with long term impacts. Indeed, a unilateral focus on mainstreaming may reflect a perception that [18] climate change impacts on infectious disease dynamics will be minimal in contrast to other drivers, or that that health-related vulnerabilities will be low [28]. For example, within Europe, only nine of 27 (33%) countries surveyed in 2010 had or were conducting a national assessment of potential health impacts of climate change [12]. The majority of experts interviewed indicated that their country had monitoring and surveillance programs capable of addressing the threats of climate change. This is particularly surprising when considering that the most emphasized recommendation in the infectious disease adaptation literature was the strengthening of current surveillance systems, a concern echoed in all national adaptation plans analyzed for the purposes of this review, and may indicate a dissonance between the spheres of public health research and practice. 

Moreover, we must note that both within the scientific literature and national adaptation planning, best practice guidelines to adapt to infectious disease risks of climate change are at the first stages of development. Indeed, few formal assessments of the feasibility and appropriateness of proposed adaptations have been conducted as of yet. However, location and sector-specific risk assessments ought to be conducted prior to the drafting of adaptation plans, in order to determine whether or not proposed adaptations will be effective in reducing climate change-induced infectious disease morbidity and mortality, and whether they truly address existing vulnerabilities [125].

Heterogeneity in national level planning for adaptation suggests that adaptation assessments will be key in guiding future adaptation policy in the coming years, by highlighting unaddressed needs, as well as gaps in problem framing. Such assessments aim to “identify modifications to current and planned programs, and opportunities for new policies and measures” [116] to ensure an optimal reduction of future infectious-disease risks. Evaluations of policy documents, such as the work presented here, can help guide the future expansion of existing adaptation planning, as well as keep climate change at the forefront of the policy debate.

## Supplementary Files

Supplementary File 1 Supplementary Information (PDF, 120 KB)
