# Supplementary material for: A Review of National-Level Adaptation Planning with Regards to the Risks Posed by Climate Change on Infectious Diseases in 14 OECD Nations
Source: Int J Environ Res Public Health. 2013 Dec 12;10(12):7083–109. doi: 10.3390/ijerph10127083 (PMC3881155; doi:10.3390/ijerph10127083)
Supplement: Supplementary File 1 — Supplementary Information (PDF, 120 KB) [file ijerph-10-07083-s001.pdf]

Supplementary Information

A Review of National-Level Adaptation Planning with Regards to the Risks Posed by Climate Change on Infectious Diseases in 14 OECD Nations

Table S1. Scientific database search strategies and number of results.

	**PubMed**	
**Set**	**Searches**	**Results**
1	(“Climate Change” [Mesh]) AND (“Communicable Diseases” [Mesh] OR infectious disease * OR vector *borne OR water *borne OR food *borne OR surveillance OR public health) AND (adapt * OR mainstream *)	510
	**Medline**	
**Set**	**Searches**	**Results**
1	climate change/ or global warming/	4,123
2	communicable diseases/ or communicable diseases, emerging/	19,596
3	disease outbreaks/ or epidemics/	61,218
4	exp Foodborne Diseases/ep, ps [Epidemiology, Parasitology]	4,449
5	Zoonoses/ or Insect Vectors/ or Disease Vectors/ or Parasitic Diseases/ or Communicable Diseases/ or Communicable Disease Control/	69,748
6	public health/ or “environment and public health”/	57,370
7	Public Health Surveillance/	86
8	2 or 3 or 4 or 5 or 6 or 7	181,135
9	Adapt *.mp.	410,871
10	Mainstream *.mp.	7,525
11	9 or 10	417,998
12	1 and 11	706
13	8 and 12	59
14	1 and 8	331
	**EmBase**	
**Set**	**Searches**	**Results**
1	climate change/	11,215
2	communicable disease/ or infection/	144,451
3	(Communicable Disease * or infectious disease * or vector * borne or water * borne or food * borne).mp. [mp = title, abstract, subject headings, heading word, drug trade name, original title, device manufacturer, drug manufacturer, device trade name, keyword]	73,621
4	public health/	75,664
5	disease surveillance/	8,289
6	2 or 3 or 4 or 5	273,681
7	(adapt * or mainstream *).mp. [mp = title, abstract, subject headings, heading word, drug trade name, original title, device manufacturer, drug manufacturer, device trade name, keyword]	336,441
8	1 and 7	1,395
9	6 and 8	157

Table S1. *Cont.*

Web of Knowledge		
**Set**	**History**	**Results**
# 5	#4 AND #3<br>Timespan = All Years<br>Search language = English	594
# 4	#2 AND #1<br>Timespan = All Years<br>Search language = English	14,505
# 3	Topic = (Communicable Disease * OR infectious disease * OR vector * borne OR water * borne OR food * borne OR surveillance OR public health)<br>Timespan = All Years<br>Search language = English	911,808
# 2	Topic = (adapt * or mainstream *)<br>Timespan = All Years<br>Search language = English	1,644,318
# 1	Topic = (climate change OR global warming)<br>Timespan = All Years<br>Search language = English	158,363
**Scopus**		
**Set**	**Searches**	**Results**
10	(TITLE-ABS-KEY (communicable disease * OR infectious disease * OR vector * borne OR water * borne OR food * borne OR surveillance OR public health)) AND ((TITLE-ABS-KEY (climate change adapt *)) OR (TITLE-ABS-KEY (climate change mainstream *)))	45
9	TITLE-ABS-KEY (climate change adapt *)) OR (TITLE-ABS-KEY (climate change mainstream *))	13,461
8	TITLE-ABS-KEY (climate change mainstream *)	369
7	(TITLE-ABS-KEY (communicable disease * OR infectious disease * OR vector * borne OR water * borne OR food * borne OR surveillance OR public health)) AND (TITLE-ABS-KEY (climate change adapt *))	44
6	TITLE-ABS-KEY (climate change adapt*)	13,224
5	((TITLE-ABS-KEY (Climate Change or Global Warming)) AND (TITLE-ABS-KEY (adapt or mainstream *))) AND (TITLE-ABS-KEY (communicable disease * OR infectious disease * OR vector * borne OR water * borne OR food * borne OR surveillance OR public health))	12
4	TITLE-ABS-KEY (communicable disease * OR infectious disease * OR vector * borne OR water * borne OR food * borne OR surveillance OR public health)	20,225
3	(TITLE-ABS-KEY (Climate Change or Global Warming)) AND (TITLE-ABS-KEY (adapt or mainstream *))	1,982
2	TITLE-ABS-KEY (adapt or mainstream *)	1,096,111
1	TITLE-ABS-KEY (climate change or global warming)	24,203

Figure S1. Peer-reviewed literature search and inclusion criteria**.**

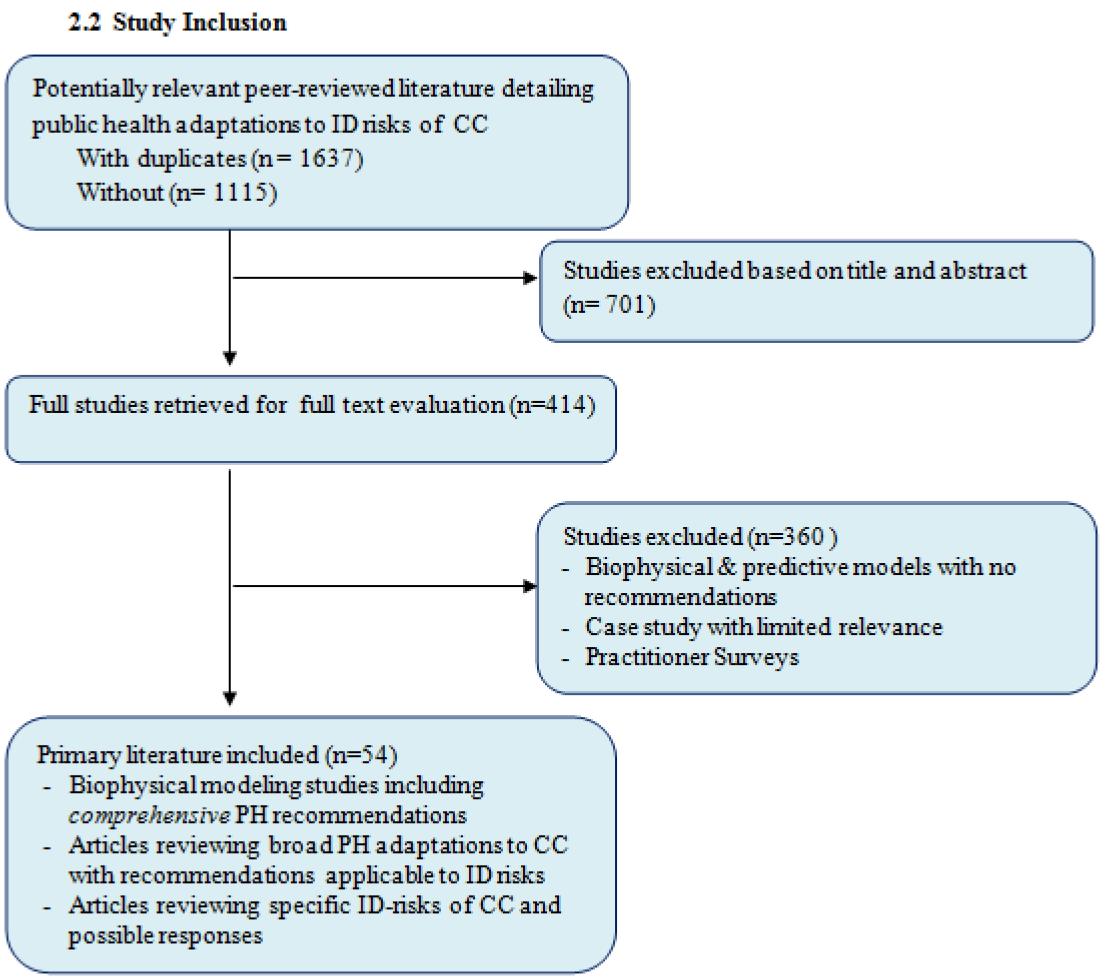

© 2013 by the authors; licensee MDPI, Basel, Switzerland. This article is an open access article distributed under the terms and conditions of the Creative Commons Attribution license (http://creativecommons.org/licenses/by/3.0/).
